# Cone-beam computed tomography-based analysis of maxillary sinus pneumatization extended into the alveolar process in different age groups

**DOI:** 10.1186/s12903-022-02445-2

**Published:** 2022-09-11

**Authors:** Xingsheng Wu, Qiudong Cai, Di Huang, Peiwen Xiong, Lianshui Shi

**Affiliations:** 1 The Affiliated Stomatological Hospital of Nanchang University, The Key Laboratory of Oral Biomedicine, Jiangxi Province, Jiangxi Province Clinical Research Center for Oral Diseases, Nanchang, 330006 China; 2 Department of Stomatology, The 908 Hospital of the Chinese People’s Liberation Army Joint Logistic Support Force, Nanchang, 330006 China

**Keywords:** Maxillary sinus pneumatization, Alveolar process, Cone-beam computed tomography (CBCT), Age

## Abstract

**Objective:**

This study aimed to measure the amount of maxillary sinus pneumatization (MSP) extended into alveolar processes in different age groups via cone-beam computed tomography (CBCT) and its association with age.

**Methods:**

The data of 293 adult patients (533 maxillary sinuses) who underwent CBCT at our hospital from January 2020 to October 2020 were analyzed and divided into the following age groups: group I (18–34 years old, youth group), group II (35–59 years old, middle-aged group) and group III (≥ 60 years old, elderly group). The distance between the lowest point of the maxillary sinus floor and nasal cavity floor in the central area of the maxillary posterior teeth was measured and recorded as the amount of MSP. Further, according to the positional relation between the maxillary posterior teeth and maxillary sinus floor, MSP was divided into type I (normal pneumatization) and type II (extensive pneumatization). The distribution of pneumatization types and degree and change of pneumatization for the different age groups were also analyzed. *P* < 0.05 was used as the threshold for statistical significance.

**Results:**

The amount of MSP of group I [(3.75 ± 3.77) mm] was significantly higher than that of group II [(2.30 ± 4.48) mm] and group III [(2.09 ± 4.70) mm], but there was no significant difference between group II and group III. We also found that the amount decreased gradually with increasing age (r_s_ = − 0.2), with the youth group showing a higher prevalence of extensive pneumatization (youth vs. middle-age vs. elderly: 66.44% vs. 36.81% vs. 22.28%, respectively). There was no statistically significant difference in the amount of MSP between males and females and between left and right maxillary sinus in each group (*P* > 0.05).

**Conclusion:**

The amount of MSP was significantly higher in the 18–34 years old group compared to older age groups, showed a decreasing trend with age and was not associated with sex and maxillary sinus sides.

## Background

With the development of modern oral implantology and the positional relationship between the maxillary sinus (MS) floor (MSF) and the root apex of the maxillary posterior teeth, MS has received increasing attention from dental practitioners. MS is fluid-filled at birth and gradually shows pneumatization with the eruption of permanent teeth. Its volume continues to expand with the bidirectional action of osteogenesis and osteoclastogenesis. However, its growth slows down with decreased facial development after puberty, but continues for a lifetime. This physiological process is called MS pneumatization (MSP) [1].

MSP can extend to adjacent anatomical structures, with the extension to the alveolar process the most common one [2, 3]. Koppe et al. found that MSP extended into the alveolar process was markedly linked to MS volume (r_s_ = 0.75) [4] and is therefore usually used to assess the overall amount of MSP.

Cone-beam computed tomography (CBCT) provides a complete and stereotactic scan around the investigated object and is widely applied in orthodontics and endodontic diagnosis [5, 6]. It is used to assess the bone density of gum, determine the height and width of alveolar bone, and in craniofacial reconstruction to measure the maxillofacial bone structure and provide fine anatomical details.

Clinical studies have found extensive MSP in young patients, making the distance between the root apex of the maxillary posterior teeth very close to the MS, which increases the risk of teeth protrusion into the sinus. Such presentations were also proved in the study by Huangdi et al. [7]. Thus, extensive MSP can lead to odontogenic sinusitis, oro-antral communication, cysts and other clinical conditions that may deeply affect the quality of life of people [8]. The purpose of this study was to measure the degree of MSP in different age groups and its changes with age, which could guide clinical procedures related to the maxillary posterior region.

## Materials and methods

### Study subjects

Adult patients for various oral reasons (implants in the anterior region, difficult cases of root canal treatment, temporomandibular disorder imaging to determine the condition of condylar bone, etc.) who underwent CBCT at the Affiliated Stomatological Hospital of Nanchang University from January 2020 to October 2020 were selected and their data were assessed. The study inclusion criteria were: (1) Age ≥ 18 years; (2) No history of head and sinus trauma and sinonasal surgery; (3) No obvious thickening of the MS mucosa and absence of MS cyst, effusion and tumor; (4) No history of MSF elevation; (5) No loss of bilateral maxillary second premolar, first molar, second molar, with good periodontal condition, and; (6) CBCT images without motion artefacts and with good differentiation between structures. Exclusion criteria: (1) history of sinus surgery; (2) history of sinus and maxillary tumors; (3) patients with cleft lip and palate.

The included patients were divided into the following groups: group I (18–34 years old, youth group), group II (35–59 years old, middle-aged group) and group III (≥ 60 years old, elderly group); based on the age classification standard of China and in combination with the age distribution of the included samples. This study was reviewed and approved by the Medical Ethics Committee of Affiliated Stomatological Hospital of Nanchang University (approval number: 2022-024).

### CBCT image acquisition and reconstruction

**Fig. 1:**
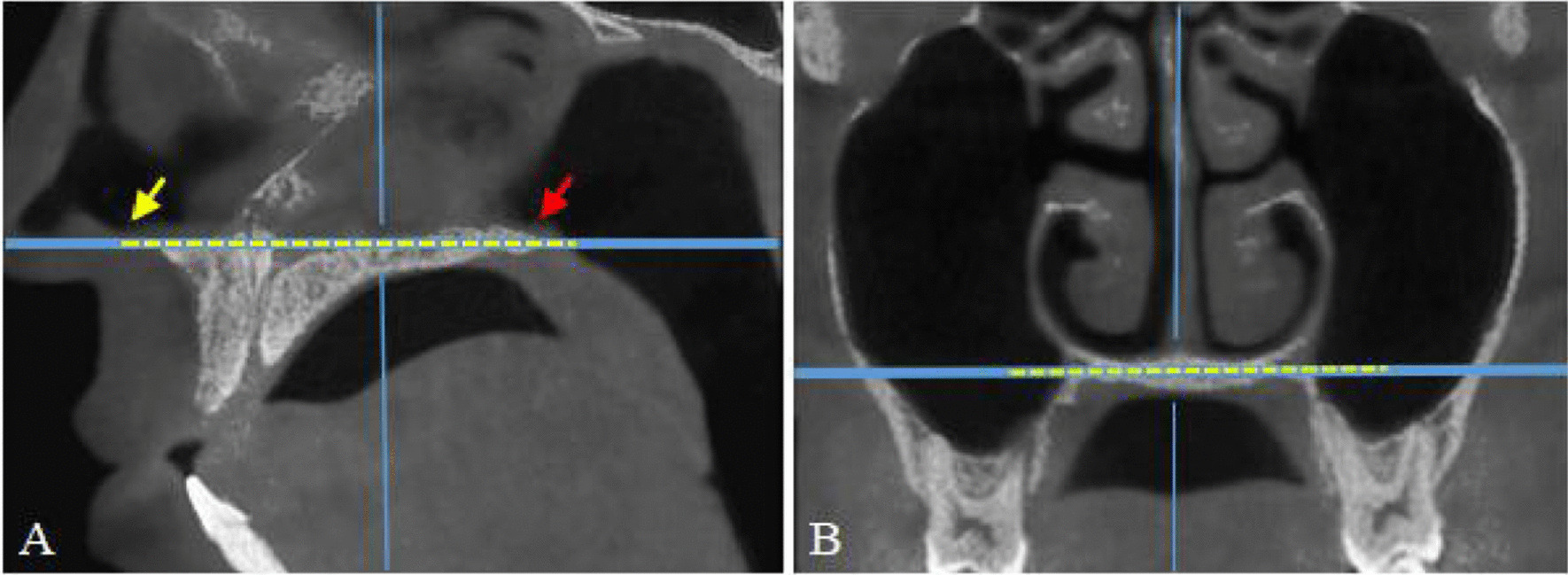
Multiplanar reconstruction of the head and maxilla. **A** Sagittal plane: the connecting line (yellow dashed line) of the anterior nasal spine (yellow arrow) and posterior nasal spine (red arrow) was parallel to the horizontal plane; **B** Coronal plane: the nasal cavity floor (yellow dashed line) was parallel to the horizontal plane

The CBCT was performed following the routine practices of the hospital. All CBCT images were obtained using the KaVo 3D eXam CBCT scanner (Kavo Company, USA), with tube voltage 120 kV, tube current 5 mA, scan time 14.7 s, voxel 0.25 mm, 16 × 13 cm field of view. Image analysis was performed using PacsView v7.0 (Tianjianyuan Biotechnology Co., Ltd., Beijing, China). In the sagittal position, the connecting line of the anterior and posterior nasal spine was chosen as the palatal plane, parallel to the horizontal plane (Fig. 1A). In the coronal reconstruction, the nasal cavity floor (NCF) was positioned parallel to the horizontal plane (Fig. 1B) to standardize the head and maxillary positions during image acquisition.

### The distance of maxillary sinus extended into alveolar process pneumatization

**Fig. 2:**
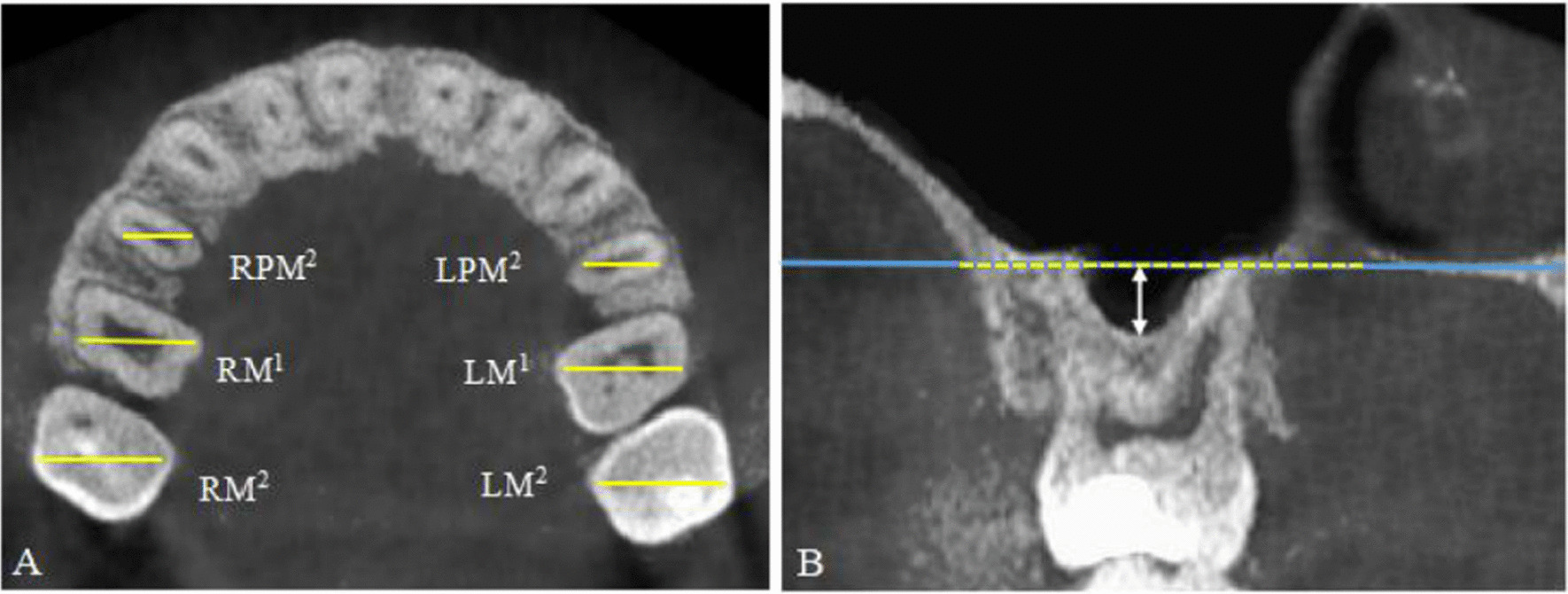
CBCT scanning area and measurement of maxillary sinus pneumatization. **A** CBCT scanning area: the central area of maxillary second premolars (LPM^2^, RPM^2^), first molars (LM^1^, RM^1^), and second molars (LM^2^, RM^2^) located at the level of the cervical region of the teeth; **B** Measurement of maxillary sinus pneumatization at RPM^2^ in a female patient: the yellow dashed line refers to the tangent of the nasal cavity floor, and the white double-headed arrow represents the linear distance from the nasal floor to the lowest level of the maxillary sinus floor

After repositioning the head and maxillary sinus, the image of the axial reconstruction was obtained, with focus on the cervical roots of the upper teeth to determine the central area of the midpoint of the mesiodistal diameter and the buccal-lingual diameter of the second premolars, first molars and second molars (Fig. 2A). The distance from the lowest level of MSF determined on a coronal plane to the NCF was defined as the amount of MSP (or MS height), based on a study by Wagner et al. [9] and Cavalcanti et al. [10] (Fig. 2B). A negative value indicated that the sinus floor was above the NCF, while a positive value suggested that the sinus floor was below the NCF.

### Classification of maxillary sinus pneumatization extension into the alveolar process

**Fig. 3:**
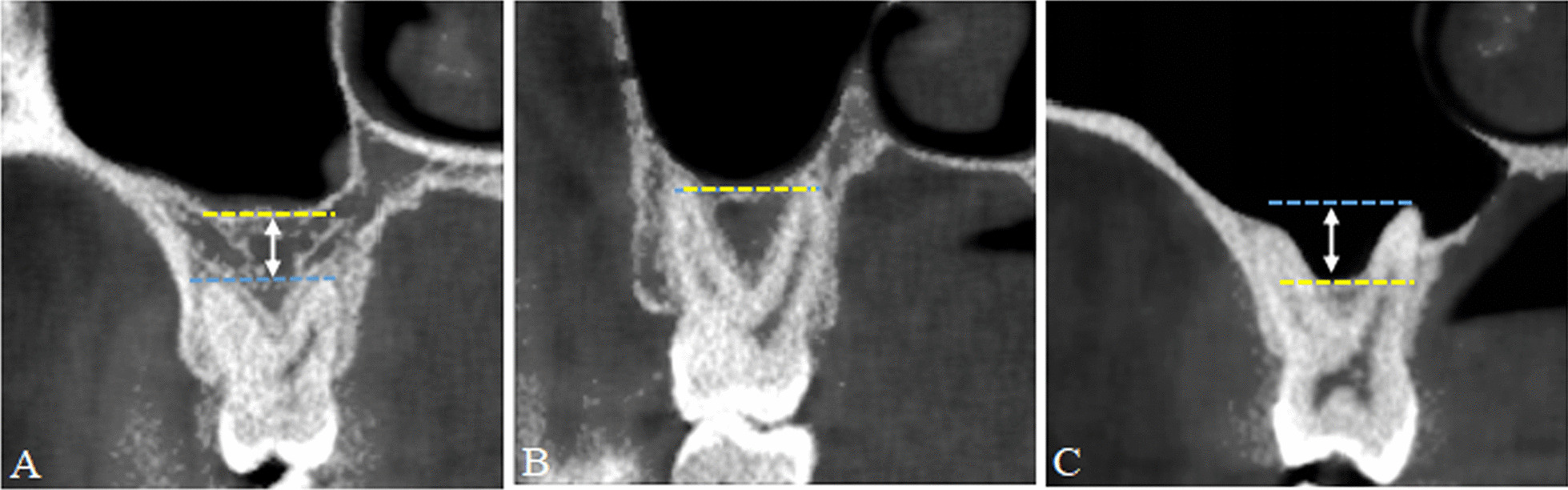
Schematic diagram of classification of maxillary sinus pneumatization. **A** Type I normal pneumatization; **B** and **C** Type II extensive pneumatization. Yellow dashed line: horizontal line at the sinus floor; blue dashed line: horizontal line at the root apex; white double-headed arrow: distance between the sinus floor and the root apex

Referring to the classification of the relationship between the MSF and the maxillary posterior teeth proposed by Sharan et al. [11] and Pei et al. [12], MSP was subdivided into the following types: type I, normal pneumatization, and type II, extensive pneumatization. In normal pneumatization, there was a certain distance between the root apex of the maxillary posterior teeth and the MSF (the MSF was apical to the level of root apex, Fig. 3A). In extensive pneumatization, the root apex of the maxillary posterior tooth was in close contact with the MSF (Figs. 3B), and the root apex was located on the medial and lateral side of the MSF or protruding into the MSF (the MSF was coronal to the apex of one of the roots, Fig. 3C). The maxillary posterior teeth corresponding to the teeth at the deepest position of the MSF was determined by the above method.

### Statistical analysis

The data were analyzed using the SPSS v26.0 statistical software (IBM). Quantitative variables are described as mean ± standard deviation (SD), and the intra-class correlation coefficient (ICC) was calculated to assess intra-observer reliability with a 95% confidence interval (CI) for measuring MSP. Additionally, a two-sample T-test, one-way analysis of variance and Tukey’s post-hoc test were used to compare the three age groups. Correlations between age and amount of MSP were examined through Pearson’s correlations, and the strength of the correlations was classified as weak (r_s_ = 0.1–0.3), moderate (r_s_ = 0.4–0.6) and or strong (r_s_ = 0.7–0.9). The qualitative variables were described using constituent ratio or rate and were interpreted by two doctors (LS Shi and XS Wu) with extensive clinical experience trained in scan reading. When the two doctors’ conclusions differed on the image results, they re-analyzed the images and discussed their results until a consensus was reached. The Chi-square test was employed for comparisons among the three age groups. *P* < 0.05 was considered statistically significant for all tests.

## Results

### Demographic data

A total of 293 patients with 533 maxillary sinuses were included in this study. The patients comprised 141 males (254 maxillary sinuses) and 152 females (279 maxillary sinuses) with an age range of 18–76 years (mean age: 38.19 ± 15.27 years).

### Comparison of the amount of maxillary sinus pneumatization extended into the alveolar process

Based on age groups, the mean age of patients from group I was 25.58 ± 0.40 years (n = 147; 289 maxillary sinuses), group II was 44.72 ± 0.70 years (n = 101; 182 maxillary sinuses), and group III was 64.71 ± 0.60 years (n = 45; 62 maxillary sinuses). Significant differences in mean age for all three age groups (*P* < 0.001). The female to male distribution in this study did not differ significantly (*P* = 0.705) (Table 1).

**Table 1:** Age and gender characteristics of the three groups

Variables	Group I (n = 147)	Group II (n = 101)	Group III (n = 45)	*P* value
Age, years (mm)	25.58 ± 0.40	44.72 ± 0.70	64.71 ± 0.60	< 0.001
Gender, n (%)				0.705
Male	68 (46.3%)	52 (51.5%)	21 (46.7%)	
Female	79 (53.7%)	49 (48.5%)	24 (53.3%)	

Age was expressed as mean ± standard deviation and one-way analysis of variance was performed for statistical analysis. Gender was expressed by n (%), and chi-square test was used to analyze statistical differences

CBCT image assessment results showed reliability of MSP values measured in 393 maxillary sinuses, with an ICC value of 0.991 (95% CI 0.984–0.995). The amount of MSP (mean ± SD) in groups I, II and III were 3.75 ± 3.77 mm, 2.30 ± 4.48 mm, and 2.09 ± 4.70 mm, respectively (Table 2). The findings showed a significant difference in the amount of MSP between groups I and II (*P* < 0.050) and I and II (*P* < 0.050), while no significant difference was found between groups II and III (*P* > 0.050).

**Table 2:** Group comparison of the amount of maxillary sinus pneumatization extended into the alveolar process

Variables	Group		Group		Group	
	I	II	I	III	II	III
Mean ± SD (mm)	3.75 ± 3.77	2.30 ± 4.48	3.75 ± 3.77	2.09 ± 4.70	2.3 ± 4.48	2.09 ± 4.70
No. of patients	289	182	289	62	182	62
Min	− 10.59	− 11.77	− 10.59	− 11.77	− 11.77	− 11.77
Max	11.48	10.72	11.48	10.72	10.72	10.72
P^*^	< 0.050		< 0.050		> 0.050	

Mean ± standard deviation, the amount of maxillary sinus pneumatization extended into alveolar process. Two-sample t-test was used for comparison between groups

**Fig. 4:**
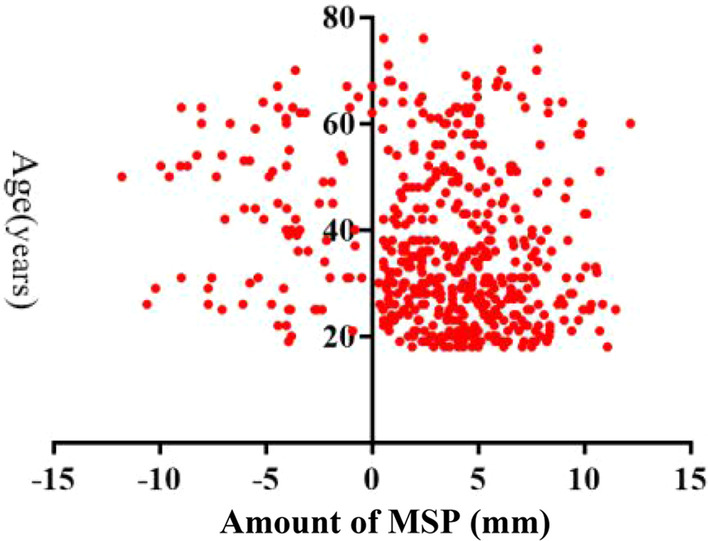
Scatter diagram showing the relation between amount of of maxillary sinus pneumatization (MSP) extended into alveolar process and age (r_s_ = − 0.2). Correlations between age and amount of MSP were examined through Pearson’s correlations

Additional assessments showed no significant difference in the amount of MSP between males and females in each group, except for group II (*P* < 0.050) (Table 3). No significant difference was also observed in the amount of MSP between the left and right MS sides (Table 3). In regard to age, we observed a correlation between age and the amount of MSP (r_s_ = − 0.2, *P* < 0.05) (Fig. 4), which indicated that the amount of MSP decreased with the increase in age.

**Table 3:** Comparison of the amount of maxillary sinus pneumatization extended into alveolar process based on gender and maxillary sinus sides of each group (mm)

Group	n	Mean ± SD (mm)	Gender		*P* value	Maxillary sinus sides		*P* value
			Men	Women		Left	Right	
I	289	3.75 ± 3.77	3.54 ± 3.76 (n = 133)	3.93 ± 3.79 (n = 156)	0.382	3.74 ± 4.12 (n = 116)	4.09 ± 3.89 (n = 116)	0.507
II	182	2.30 ± 4.48	3.00 ± 4.32 (n = 95)	1.52 ± 4.56^*^ (n = 87)	0.026	1.78 ± 4.81 (n = 69)	1.84 ± 4.81 (n = 69)	0.942
III	62	2.09 ± 4.70	2.95 ± 3.52 (n = 26)	1.47 ± 5.36 (n = 36)	0.224	1.10 ± 4.79 (n = 16)	0.63 ± 5.21 (n = 16)	0.792

^*^The amount of maxillary sinus pneumatization extended into alveolar process was significantly different between males and females in group II (*P* < 0.05); Mean ± standard deviation, the amount of maxillary sinus pneumatization extended into alveolar process

### Comparison of the composition of pneumatization types among different age groups

**Fig. 5:**
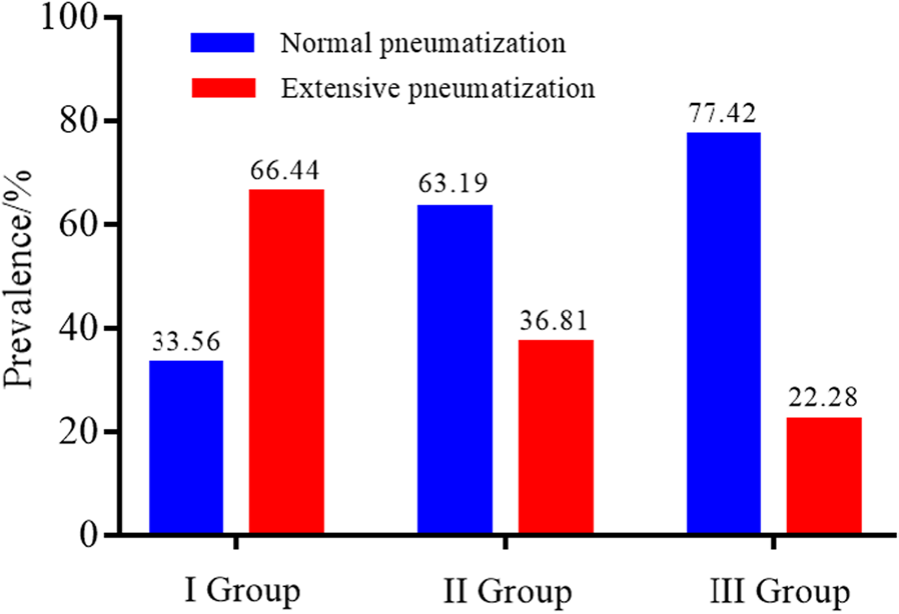
Classification of maxillary sinus pneumatization in different age groups

Here, we divided the degree of MSP in each group into two types: normal pneumatization and extensive pneumatization. Our findings showed that the ratios of extensive and normal pneumatization in group I were 66.44% and 33.56%, group II, 36.81% and 63.19%, and group III, 22.28% and 77.42%, respectively. Among them, the highest constituent ratio of extensive pneumatization was found in group I (Fig. 5).

## Discussion

This study investigated the amount and degree of MSP with age, gender and MSP sides in a cohort of 293 patients, comprising a total of 533 maxillary sinuses. The 18–34, 35–59 and ≥ 60 age groups were selected to represent the young (group I), middle-aged (group II) and elderly groups (group III), respectively. Although the amount of maxillary sinus pneumatization extended into the alveolar process was significantly different between males and females in group II, overall, no significant difference was observed in terms of gender and MSP sides. The amount of MSP between groups I and II and I and II was significantly different, while no significant difference was found between groups II and III. Pearson’s correlations showed a weak correlation between age and the amount of MSP, indicating that the amount of MSP gradually decreased with increasing age.

Pneumatization starts from birth and tends to be stable in adulthood [13], which could explain the high rate of extensive pneumatization observed in our study in the young age group. It was reported that the incidence of extensive MSP in adolescents could be as high as 81.08% [7]. In our study, the rate of extensive pneumatization was highest in the 18–34 years old group (group I vs. II vs. III: 66.44% vs. 36.81% vs. 22.28%). This can be explained by the improvement in height and body mass index of the younger generation. Further, differences in living habits and dietary structure compared to the older generation might be a potential cause. For instance, young people nowadays consume more processed food due to their busy routine and less time to cook. Such food usually requires less occlusal force and chewing, reducing the number of functional stimulation during jaw development and thus potentially inducing extensive MSP in younger people. Extensive MSP is associated with endodontic and periodontal diseases, whereby the teeth surpass the teeth-supporting tissues and enter the MS, causing odontogenic maxillary sinusitis [14]. Sharan et al. found that secondary MSP occurred more easily after tooth extraction in patients with extensive pneumatization, and such change greatly reduced the height available for implant placement [15]. Thus, related authorities should be concerned about this condition in the younger population, and strategic measures should be implemented after further validating these observations via population-wide assessments.

Bornstein et al. [16], Luz et al. [17] and Cavalcanti et al. [10] reported a statistical difference in the MSP degree between males and females, with males having greater MS volume than females. Further analysis showed that the difference might be due to the larger proportion of skulls and bodies and greater MSP degree in males. However, Takahashi et al. [18] indicated that although MS volume decreased with age, there was no significant difference between males and females, which could be explained due to the reduced gender in the investigated study cohort. Aktuna Belgin et al. [19] reported that MS volume was notably greater in men than in women in the age group of 18–24 years old but was not significantly different from age ≥ 25 years old. This was consistent with the results of Jun et al. [13], who reported that the development of MS continued until the second and third decades of life in women and men, respectively. In this present study, we observed no statistical difference in the amount of MSP within group I (mean age of 25.58 ± 0.40 years) and III (mean age of 64.71 ± 0.60 years), while in group II a notable difference was observed, with men having greater amount of MSP than women in the middle-aged population. We hypothesized that this could have been caused due to the irregular ever-changing shape of MS despite the volume being almost similar [20] or possibly the presence of other unaccountable factors. Further, we also found no significant difference in the amount of MSP between left and right MS sides within each group, which was in agreement with some previous studies [10, 16, 17], and the exact cause remains unknown.

This study has some limitations. First, the CBCT images were retrospectively collected from patients who came to the hospital for treatment of various causes without detailed information about their ethnicity, medicine use, drug dosage, etc. Second, the study lacked consideration of the changes in individual MSP with age, and we did not account for MS volume based on the difference between left and right MS sides and inter-individual errors and differences. Thus, the findings of this study should be further validated in a larger prospective cohort and consider to these limitations.

## Conclusion

Overall, the amount of MSP was significantly associated with patients’ age, showed a decreasing trend with increasing age and was more prevalent in the 18–34 years old group, while it was not significantly associated with gender and MS sides. Therefore, considering the consequences of MSP extended into the alveolar process, it could be important to determine the amount and degree of MSP before treatment of the maxillary posterior region, especially in high-risk patients, such as those aged 18–34 years old.

## Data Availability

The datasets used and/or analyzed during the current study are available from corresponding author on reasonable request.

## Abbreviations

MSP: Maxillary sinus pneumatization
CBCT: Cone beam computed tomography
NCF: Nasal cavity floor
ICC: Intra-class correlation coefficient
CI: Confidence interval
